# Growth data and tumour risk of 32 Chinese children and adolescents with 45,X/46,XY mosaicism

**DOI:** 10.1186/s12887-019-1520-9

**Published:** 2019-05-06

**Authors:** Lili Pan, Zhe Su, Jianming Song, Wanhua Xu, Xia Liu, Longjiang Zhang, Shoulin Li, on behalf of the multidisciplinary collaboration team of DSD management at Shenzhen Children’s Hospital

**Affiliations:** Director of Endocrinology department, Shenzhen Children’s Hospital, No. 7019, Yitian Road, Shenzhen, 518038 Guangdong Province People’s Republic of China

**Keywords:** 45,X/46,XY mosaicism, Growth, Gonadoblastoma, Gonadal dysgenesis

## Abstract

**Background:**

The aim of this study was to review the growth data, gonadal function and tumour risk of children and adolescents with 45,X/46,XY mosaicism who presented to a single centre in China.

**Methods:**

We conducted a retrospective review of the records of 32 patients with 45,X/46,XY mosaicism or variants who were hospitalized from August 2005 to September 2018. The main outcomes measured were growth data, genital phenotype, gonadal function, gonadal position, and histological results.

**Results:**

A total of 32 patients were included. The age at diagnosis ranged from 0.6 to 16.3 years. Nineteen patients exhibited ambiguous genitalia, 12 had short stature, and 1 showed a lack of breast development. Seventeen patients were raised as males, and 15 were raised as females. The external masculinisation score (EMS) of patients raised as male was 4.5 (1~12) [median (range)]. The EMS of the females was 0 (0~1.5) [median (range)]. Patients showed normal heights under 2 years old, with a height SDS of 0 (− 1.5~1.4) [median (range)]. Growth appeared to decelerate after age 2 years, with SDS decreased to − 2.8 (− 3.0~ − 0.9) [median (range)]. The percentage of short stature was higher in females than in males (76.9% vs 50.0%). Twenty-five patients had gonadal pathological results. Complete gonadal dysgenesis (CGD) and mixed gonadal dysgenesis (MGD) were the most common pathogenic subtypes, accounting for 48.0 and 36.0%, respectively. Ovotesticular tissue was observed in only 4.0% of patients. Gonadoblastoma and positive OCT3/4 results were found in 18.8% of gonads in children over 2 years of age. Palpable gonads accounted for 50% of these. All patients who had gonadoblastoma were raised as females.

**Conclusions:**

Patients with 45,X/46,XY might have normal heights until 2 years old.

Growth decelerations after 2 years of age were common. Patients who are being raised as females seemed to be shorter than males. CGD and MGD were the most common gonadal pathogenic subtypes. The tumour risk is high in these patients, even in palpable gonads and female patients.

## Background

The 45,X/46,XY disorders of sex development (DSD) is a rare congenital malformation [1, 2]. It occurs with an estimated incidence of 1 per 10,000 individuals [3]. Only approximately 10 papers have focused on children and adolescent patients. There have been limited reports of 45,X/46,XY mosaicism from China, and few of these include follow-up data on the patients’ heights or the risk factors for gonadoblastoma. In the current study, we identified 32 children and adolescents with 45,X/46,XY mosaicism and reported their growth data, genital phenotypes, gonad function evaluation, gonadal position and histological results, as well as a review of the literature.

## Methods

### Patients

In this retrospective analysis, we identified all patients with 45,X/46,XY mosaicism who were hospitalized in Shenzhen Children’s Hospital from August 2005 to September 2018. We also included patients who showed aberration of the Y-chromosome. We evaluated 44 patient record files, of which 12 had incomplete information and were eliminated. A final group of 32 consecutive patients were included in the study.

### Karyotyping

Blood sample (0.3–0.5 ml, heparin anticoagulation) was added into cell culture medium (Dubai Biomedical Co. Ltd., Guangzhou, China). Dolchicine was added into the culture medium at 69 h. Culture was carried out for 72 h. Karyotyping were performed on cultured blood lympthocytes arrested in the mitosis phase and stained with Giemsa dye. Thirty to 100 mitoses were examined to determine the percentage of cell line mosaicism. All karyotypes were evaluated by an experienced clinical geneticist, and according ISCN 2016 [4].

### Clinical examinations

Patients’ heights were measured on a wall-mounted stadiometer. Height evaluation was calculated as described by Hu et al. [5], and is expressed as age- and sex-specific standard deviatibon scores (SDSs). Pubertal staging was performed according to the criteria of Tanner [6, 7]. Testicular volume was measured using a Prader orchidometer. Spontaneous pubertal onset was defined as testicular volume ≥ 4 ml for males or the appearance of breast stage 2 for females without sexual hormone replacement. All patients were scored using the external masculinisation score (EMS) as described by Ahmed et al. [8]. The hypothalamic-pituitary-gonadal (HPG) axis function was evaluated by basal sexual hormone examination, gonadotropin releasing hormone (GnRH) stimulating assay (injectable gonadorelin 2.5–3.0 μg/kg and no more than 100 μg at a time), and human chorionic gonadotrophin hCG stimulation test (a single dose of hCG injection at a dose of 500 IU/Day, 1000 IU/Day and 1500 IU/day × 3 or every other day× 3 for patients younger than 1 year old, between 1 to 10 years old and older than 10 years, respectively). Hypergonadotropic hypogonadism was defined as basal follicle stimulating hormone (FSH) greater than or equal to 40 IU/L [9].

### Surgery and histology

Before surgery, decisions were considered by a multidisciplinary team, including endocrinologists, urologists, gynaecologists, psychologists, pathologists, and ethics committees, and were discussed with the family at all stages of this process.

Gonad tissue samples were fixed in buffered formalin. Histological examinations were performed on haematoxylin-eosin-stained sections. Complete gonadal dysgenesis (CGD) was defined as bilateral streak gonads. Partial gonadal dysgenesis (PGD) was defined as dysplastic testes. Mixed gonadal dysgenesis (MGD) was defined as a streak gonad on one and a contralateral testis. Ovotesticular tissue (OT) was defined as the combined presence of testis and ovarian tissue in the same individual [10].

Immunohistochemical evaluation with antibodies against octamer binding transcription factor 3/4 (OCT3/4) [11] was performed in all gonadal tissues, using the following antibody anti-OCT3/4 (MAB-0618, Fuzhou Maixin Biotech. Co. Ltd., China). A standard indirect peroxidase method with reagents and secondary antibodies from the Ultra-view Universal Diaminobenzidine (DAB) kit (Dako, Denmark) was provided by Roche. DAB was used as chromogen in the peroxidase staining for OCT3/4. Positive results were evidenced by brown staining. The positive control was sample from germinoma which is known as OCT3/4 positive. The negative control used the patient sample with a primary antibody replaced by Phosphate Buffered Saline (PBS).The description of the gonadal pathology and tumours was performed according to WHO Classification of Tumours of the Urinary System and Male Genital organs [12].

### Literature review and search strategy

We searched two databases, PubMed and China National Knowledge Infrastructure (CNKI), for articles published from January 1988 to September 2018 using the following keyword: 45,X/46,XY mosaicism, growth and gonadal dysgenesis. Reports with more than 7 cases were included.

## Results

Karyotypes of all the patients were 45,X/46,XY. However, three of them (No. 3, 5 and 21) had variants of the 45,X/46,XY karyotype: 45,X/46,XY/46,X,i(Y)(q10), 45,X/47,XYY and 45,X/47,XXY.

The ages at diagnosis ranged from 0.6 to 16.3 years. The patients came with the following main complains: 19 presented with ambiguous genitalia, 12 exhibited short statures and 1 showed lack of breast development at age 14.3 years. Among the 32 patients, 17 patients were raised as males and 15 were raised as females. The EMS of patients raised as male was 4.5 (1~12) [median (range)]. The EMS of the females was 0 (0~1.5) [median (range)]. Seven out of 13 patients were consistent with the phenotypical features of Turner syndrome, such as widely spaced nipples and hypoplastic nails. Five patients (5/32, 15.6%) had congenital abnormalities, including horseshoe kidneys, ventricular septal defect, and duplex kidney. One patient had neural hearing loss and the phenotypical features of Turner syndrome.

### Growth data

**Fig. 1:**
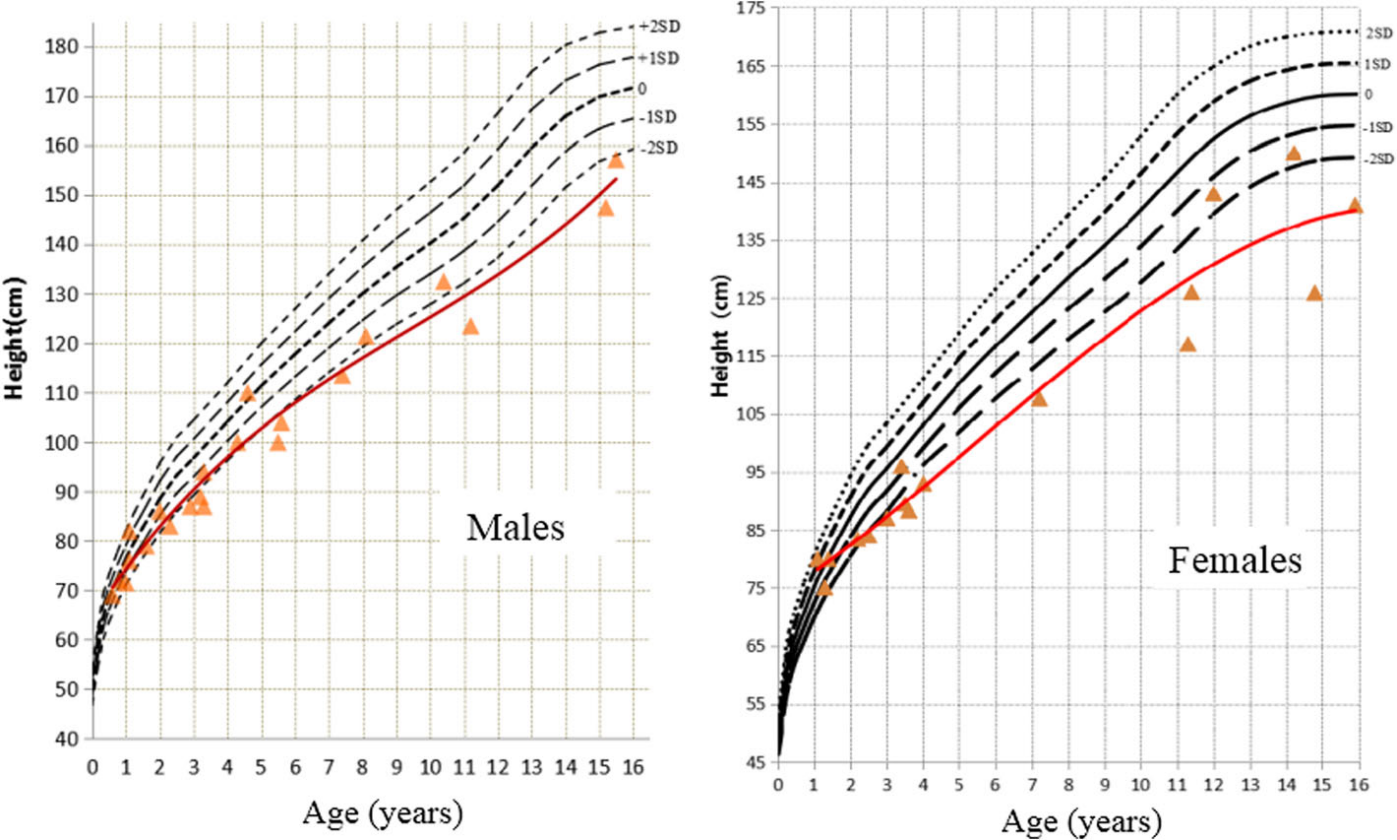
Heights of patients. Heights were within the normal range in 7 patients who were measured before 2 years of age. Those patients revealed growth deceleration after the age of 2 years. Seventeen out of 27 patients (17/27) who were older than 2 years presented short stature (less than − 2 SDS). The percentage of patients with short stature was higher in females than in males

In our study, there were 27 patients with records of height. Heights measured before 2 years of age (*n* = 7) were all within normal range, with a height SDS of 0 (− 1.5~1.4) [median (range)]. Heights measured after 2 years of age (*n* = 27) were much shorter than normal, with a height SDS of − 2.6 (− 6.0~0.5) [median (range)]. Among those 27 patients, 17 presented short stature (less than − 2.0 SDS). The percentage of short stature was higher but not significantly in females than in males [76.9% (10/13) vs 50.0% (7/14), *P* = 0.2943]. The shortest patient in our study was a 14.8-year-old girl whose height was − 6.0 SDS (Fig. 1). Interestingly, follow-up of those 7 patients who had height records before 2 years of age revealed growth deceleration after the age of 2 years. The height SDS decreased to − 2.8 (− 3.0~ − 0.9) [median (range)] (Fig. 1). The changes in height SDS were − 1.5 (− 0.7~ − 4.2) [median (range)].

### Pubertal development and HPG axis evaluation

Two out of 32 patients (No. 1 and 27) had spontaneous pubertal development with testicular volumes of 8 ml at age 12.4 years and 6 ml at age 15.2 years, and the EMSs were 4.5 and 12, respectively. Their LH levels increased from 4.66 IU/L to 51.37 IU/L and from 2.18 IU/L to 33.92 U/L, and their FSH levels increased from 14.53 IU/L to 30.48 IU/L and from 6.97 IU/L to 17.31 IU/L on the GnRH stimulating test, respectively.

The other nine patients (9/24) (No. 13, 14, 15, 19, 20, 22, 29, 24 and 31) met the diagnosis of hypergonadotropic hypogonadism according to basal FSH levels, except for No. 24 and No. 31, whose basal FSH levels were 33.61 IU/L and 35.71 IU/L at the age of 3.5 and 4.0 years, respectively. The gonadal pathologies of those two patients were CGD.

GnRH stimulation tests were performed in 13 other patients. Their peak LH were all more than 1 IU/L. HCG stimulation tests were performed in twelve out of those 13 patients. Seven patients showed an increase in serum testosterone to more than 1 ng/ml (No. 6, 10, 11, 17, 21, 25 and 26), while 5 patients (No. 4, 8, 12, 30 and 32) failed to increase. The median EMSs of the groups of patients were 4.5 and 1.25, respectively.

### Surgery and histological findings

Twenty-five patients underwent gonadal biopsy, and 13 out of 25 patients underwent bilateral gonadectomy. Two male patients (No. 8 and 9) underwent bilateral gonadectomy at 2.7 and 1.4 years, and another 11 female patients underwent bilateral gonadectomy (No. 13, 14, 15, 16, 19, 20, 24, 28, 29, 31 and 32) at the ages of 16.5, 11.4,14.5, 10.3, 16.2, 12.0, 3.6, 3.3, 4.0 and 8.0 years, respectively.

Thirty out of 32 patients were assigned to their previous gender, 2 patients (No. 8 and 9) underwent gender change from male to female at the ages of 2.7 and 1.6 years.

The gonadal pathogenic results were related to gonad positions. For 16 palpable gonads, (14/16) were dysgenetic testis, whereas 91.2% (31/34) of impalpable gonads were streak gonads. CGD and MGD were the most common gonadal pathogenic subtypes in our study. Twelve patients (12/25, 48%) presented CGD, all of whom were raised as females. Nine patients (9/25, 36%) showed MGD, seven of whom were raised as males and 2 as females. Three male patients (3/25, 12%) presented with PGD. One female patient (1/25, 4%) showed OT.

Overall, three out of 25 patients (No. 24, 28 and 32) had gonadoblastoma in 3 gonads. Diagnoses were made at the ages of 3.5, 3.3 and 8.0 years. The percentage of patients with gonadoblastoma reached 27.3% (3/11) of the female phenotype (EMS = 0). Two patients (No. 2 and 18) with 3 gonads had positive reactions for OCT3/4 at the ages of 4.6 and 11.3 years, respectively. Gonadoblastoma and positive OCT3/4 results were found in 18.8% (6/32) of gonads in children over 2 years of age. Those positive results encompassed 6 gonads, with the locations being in the scrotal fold (2/6), inguinal region (1/6) and intra-abdominal region (3/6). Palpable gonads accounted for 50% of them.

**Fig. 2:**
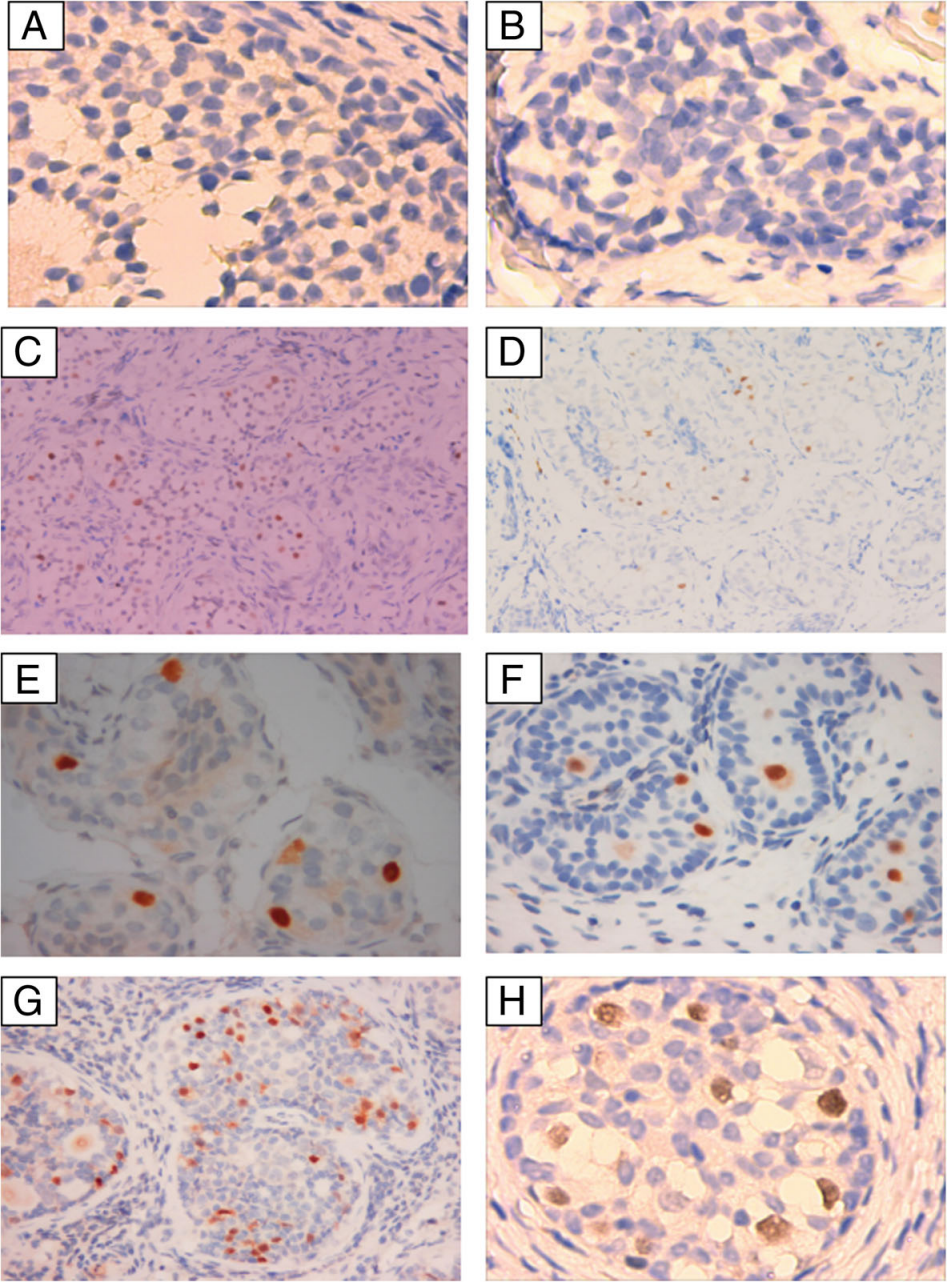
Results of immunohistochemistry staining. A and B (No. 21 and 31), negative for OCT3/4(× 400); C~H (No. 2, 9,18, 26, 28 and 32), OCT3/4-positive staining (brown nuclear signal, A, B, E and H × 400, C and D × 100, F and G × 200)

**Fig. 3:**
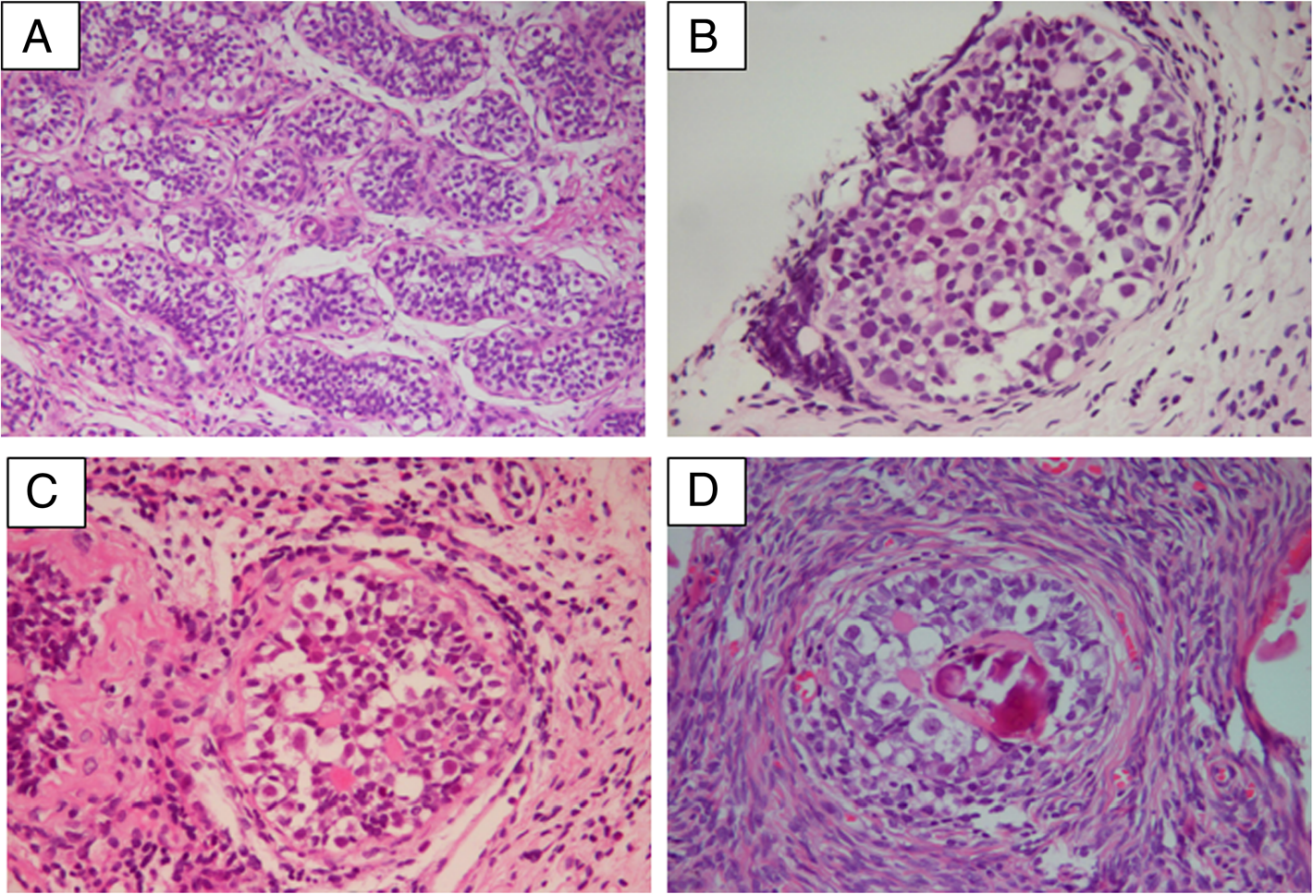
Histological examination of gonadoblastoma. A, Normal testicular tissue (HE, × 100). B, C and D, gonadoblastoma (No. 24, 28 and 32, HE, × 100). The tumour cells are round or ovoid in shape and form nests that vary greatly in size. The nests are surrounded by fibrous connective tissue and have distinct borders, and calcification is present

Immunohistochemistry results and gonadoblastoma images are shown in Fig. 2 and Fig. 3, respectively.

### Literature search results

We included 17 articles, of which 15 were written in English and 2 in Chinese; at the same time, we also read the review article by Colindres [13]. The characteristics of included studies are summarized in Table 1.

**Table 1:** Summary of the literature review

Year	Nation	Author	Cases	NO. < 18 Y	Other congenital abnormalities	Height <− 2.0 SDS	Gonadal histology
1990	USA	Chang3	76	/	5	/	OT2/3;PTL 1/3
1999	France	Telvi16	27	27	8	23	CGD9/27,PGD7/27,MGD11/27
2005	Greece	Patsalis23	11	/	4	/	CGD8/11,PGD1/11,MGD2/11
2009	USA	Kibar24	14	14	2	/	CGD2/9, MGD7/9
2010	USA	Tosson17	16	16	/	8	CGD3/15,PGD4/15, MGD9/15;GB2/15
2012	France	Martinerie18	20	20	9	14	PGD1/18,MGD17/18
2012	Denmark	Lindhardt22	25	15	3	/	CGD3/15,PGD5/15,MGD6/15,OT1/15
2012	Turkey	Ocal10	11	11	/	/	CGD2/11,PGD4/11,MGD4/11,OT1/11
2013	UK	Farrugia25	31	31	15	/	/
2014	Brazil	Rosa15	14	12	4	6	/
2015	China	Chen19	7	7	/	7	/
2015	USA	Dendrinos28	16	16	/	/	GB4/16
2016	China	Jiang26	32	/	3	/	CGD26/31,PGD5/31;GB4/Seminoma1(5/32)
2016	China	Tam29	21	21	/	/	CGD9/20,PGD11/20; CIS + GB 9/20
2017	China	Wu20	16	5	1	9	CGD2/2
2018	Iran	Mohammadpour30	49	/	/	/	PGD11/11
2018	China	Huang21	19	19	5	15	CGD11/18,PGD2/18,MGD5/18;GB2/18
			405	214	59 (59/285)	82	CGD75/191,PGD51/191, MGD61/191,OT4/191;
						(82/119)	GB + CIS + PLT + Seminoma21/104
2018	China	Our study	32	32	5 (5/32)	17 (17/27)	CGD12/25,PGD3/25,MGD9/25,OT1/25; GB3/25

*Y* years; *CGD* complete gonadal dysgenesis; *PGD* partial gonadal dysgenesis; *MGD* mixed gonadal dysgenesis; *OT* ovotesticular tissue; / not specified;

*PTL* precancerous testicular lesion; *GB* gonadoblastoma; *CIS* carcinoma-in situ

## Discussion

The possible mechanism of 45,X/46,XY mosaicism is thought to be the loss of non-disjunction of the Y chromosome after normal disomic fertilization [14]. 45,X/46,XY mosaicism can present with a wide spectrum of phenotypes in different age groups. Chang et al. suggest that 95% of X/XY fetuses will have normal male genitalia [3]. However, most patients who were diagnosed during infancy had ambiguous genitalia. Adolescent patients may present with a lack of puberty signs, leading to infertility in adulthood [15].

In our study, the second most common complaint was short stature. Heights seemed to be normal under the age of 2 years, but growth deceleration was found thereafter. Among patients older than 2 years, 62.9% presented short stature. Females seemed to be shorter than males were, though not significantly. The shortest girl was slightly taller than the previously reported shortest female (− 6.0 SDS vs − 7.0 SDS) [16]. Similar to the data found in the literature review [15–21], more than half of the patients had short stature without growth hormone treatment [82/119 (68.9%)]. Normal heights under age 2 years cannot predict normal stature later in life. Girls may need closer follow-up and more aggressive intervention. Notably, published evidence suggests that growth hormone treatment may be beneficial [17, 18, 22], with prophylactic gonadectomy being advocated prior to such treatment.

In our study, 40.6% patients exhibited the phenotype of Turner syndrome and/or other congenital abnormalities, and they were all raised as females, whereas 15.6% of patients had other congenital abnormalities. A literature review of 285 cases with phenotype descriptions showed similar proportions of patients with additional congenital abnormalities of 59/285 (20.7%) [3, 15, 16, 18, 20–26] (Table 1). Notably, compared to males, subjects assigned to the female gender showed a higher incidence of other congenital abnormalities [27]. Such patients should undergo a comprehensive assessment similar to that for Turner syndrome.

45,X/46,XY mosaicism used to be also called MGD or ovotesticular DSD. However, in our CGD and MGD were the most common gonadal pathogenic subtypes with proportions of 48 and 36%, respectively. The other two subtypes accounted for less than 20% of subjects. The literature review revealed similar percentages [3, 10, 16–18, 20–24, 26, 28–30]: CGD in 79/191 patients (41.4%), MGD in 61/191 (31.9%), PGD in 51/191 (26.7%) and OT in 4/191 (2.1%) patients (Table 1). More patients with a female phenotype or severe under virilisation phenotype tend to require a gonadal biopsy. This may be one possible reason for the higher proportion of patients with CGD in our study than that observed previously.

There have been a few reports on gonadal tumour risks in children. In our study, the total percent of gonadoblastoma and positive OCT3/4 was 21.7%, similar to that found in the literature review, which was 20.2% (21/104) (Table 1). Immature germ cells are immunohistochemically characterized by increased TSPY expression and prolonged expression of embryonic germ cell markers, including OCT3/4 [31]. The combination of OCT3/4 and TSPY expression is considered valuable for the identification of malignant germ cells in dysgenetic gonads [29, 32–34]. Owing to the unavailability of other biomarkers such as TSPY, the risk of tumours in our study may have been underestimated. However, clinicians need to pay attention to the age-related change in OCT3/4, as OCT3/4-positive germ cells in patients under 2 years old indicates delayed maturation [35]. In our study, tumour risk seemed high even in palpable gonads (50%), which was also higher than the values observed in Cools and Tam’s reports [35.7% (5/14) and 25% (3/12), respectively] [29, 31]. Tumour risk in patients with a female phenotype was 13%, which is less than the 52% risk in patients with an ambiguous phenotype (0 < EMS < 7.0) [32]. However, in our study, 27.3% of female phenotype patients had gonadoblastoma. Tam et al. [29] also reported that 6/11 (54.5%) patients with female phenotype or raised as a female had gonadoblastoma or carcinoma-in situ. Therefore, the tumour risk may be high even in palpable gonads and in female patients. Gonadal biopsy is necessary without delay.

## Conclusions

Patients with 45,X/46,XY mosaicism might have normal heights under the age 2 years. Growth decelerations after 2 years of age were common. Patients raised as females seemed to be shorter than males. CGD and MGD were the most common gonadal pathogenic subtypes. The tumour risk might be high, even in palpable gonads and female patients.

## Abbreviations

CGD: Complete gonadal dysgenesis
DSD: Disorder of sex development
EMS: External masculinization score
FSH: Follicle stimulating hormone
GnRH: Gonadotrophin-releasing hormone
hCG: human chorionic gonadotrophin
HPG: Hypothalamic-pituitary-gonadal
LH: Luteinizing hormone
MGD: Mixed gonadal dysgenesis
OCT3/4: Octamer binding transcription factor 3/4
OT: Ovotesticular tissue
PGD: Partial gonadal dysgenesis
PLAP: Placental alkaline phosphatase
SDS: Standard deviation score
TSPY: Testis-specific protein on the Y chromosome
